# A Protocol for Quantifying Lipid Peroxidation in Cellular Systems by F2-Isoprostane Analysis

**DOI:** 10.1371/journal.pone.0080935

**Published:** 2013-11-14

**Authors:** Christiaan F. Labuschagne, Niels J. F. van den Broek, Pjotr Postma, Ruud Berger, Arjan B. Brenkman

**Affiliations:** 1 University Medical Center Utrecht, Department of Metabolic Diseases and Netherlands Metabolomics Center, Utrecht, The Netherlands; 2 Leiden University, Leiden Academic Centre for Drug Research and Netherlands Metabolomics Center, Leiden, The Netherlands; UAE University, Faculty of Medicine & Health Sciences, United Arab Emirates

## Abstract

Cellular systems are essential model systems to study reactive oxygen species and oxidative damage but there are widely accepted technical difficulties with available methods for quantifying endogenous oxidative damage in these systems. Here we present a stable isotope dilution UPLC-MS/MS protocol for measuring F2-isoprostanes as accurate markers for endogenous oxidative damage in cellular systems. F2-isoprostanes are chemically stable prostaglandin-like lipid peroxidation products of arachidonic acid, the predominant polyunsaturated fatty acid in mammalian cells. This approach is rapid and highly sensitive, allowing for the absolute quantification of endogenous lipid peroxidation in as little as ten thousand cells as well as damage originating from multiple ROS sources. Furthermore, differences in the endogenous cellular redox state induced by transcriptional regulation of ROS scavenging enzymes were detected by following this protocol. Finally we showed that the F2-isoprostane 5-iPF_2α_-VI is a metabolically stable end product, which is excreted from cells. Overall, this protocol enables accurate, specific and sensitive quantification of endogenous lipid peroxidation in cellular systems.

## Introduction

Reactive oxygen species (ROS) are formed during normal cellular metabolism and it is becoming increasingly apparent that they have an important, yet complicated role in biology and pathology [1,2]. The mitochondria are the main source of ROS production during the process of oxidative phosphorylation [3]. Other sources of ROS include fatty acid oxidation in the peroxisomes and enzyme complexes like NADPH oxidase [4]. Superoxide (•O_2_^-^), the primary ROS, can be further reduced to hydrogen peroxide (H_2_O_2_) and the hydroxyl-radical (OH^•-^) in the cell. As a result of the high reactivity of these molecules, they readily react with DNA, proteins and lipids to cause oxidative damage, thereby altering their function towards pathology. In addition to toxicity, it has recently become clear that ROS and even ROS damage can act as secondary messengers in signal transduction in important metabolic pathways [5-7]. 

To control ROS, cells are equipped with specific defence and repair mechanisms to ensure cellular survival. Superoxide dismutases (SOD) are the main scavengers of •O_2_^-^, reducing it to H_2_O_2_, which in turn is scavenged by catalases and glutathione peroxidases. Peroxiredoxins are important organic hydroperoxide scavengers and play a crucial role in redox signalling [8,9]. ROS are also scavenged by small molecules, including GSH, ascorbic acid and α-tocopherol [8] 

The most challenging aspect of studying ROS metabolism is the fact that they are extremely reactive and short-lived molecules, which make them difficult to measure. Various approaches have been developed for cell-based systems to measure ROS and ROS damage, but many suffer from a lack of specificity, linearity or detailed method characterization.

 Isoprostanes are chemically stable prostaglandin-like lipid peroxidation products that are endogenously formed from oxidative damage to polyunsaturated fatty acids (PUFA’s) [10]. These molecules have been used as markers for oxidative stress in human pathologies and are considered to be the gold standard in measuring systemic lipid peroxidation in mammalian plasma and urine [11]. Recently, F3-isoprostanes have been identified [12] and were used as sensitive endogenous markers of oxidative damage in *C. elegans* [2]. Although various GC- and LC-MS methods exist for F2-isoprostane measurement, these are all optimized for analysis in mammalian plasma and/or urine and do not address F2-isoprostane measurement in cellular systems. 

Here we characterize a liquid chromatography mass spectrometry isotope dilution based protocol optimized to quantify F2-isoprostanes in cellular systems and show that they are reliable markers of lipid peroxidation from various ROS sources. The developed protocol is rapid, lacks derivatization and is highly sensitive and linear over a wide dynamic range, allowing for absolute quantification of endogenous lipid peroxidation in as little as ten thousand cells. Furthermore, although some F2-isoprostanes have been shown to be further metabolized, we demonstrate that the F2-isoprostane 5-iPF_2α_-VI is highly stable and excreted from cells. Finally, this F2-isoprostane approach showed high sensitivity, which allowed for the assessment of the endogenous redox changes caused by activation of a Forkhead transcription factor, which regulates transcription of ROS scavenging enzymes. This protocol for measuring cellular lipid peroxidation by F2-isoprostane analysis may be an important tool to contribute to a better understanding of ROS metabolism in cell based systems.

## Materials and Methods

### Reagents

Synthetic 8-isoPGF_2α_-III; 8-isoPGF_2α_-III-d4; 5-iPF_2α_-VI; 5-iPF_2α_-VI-d11; 8,12-iPF_2α_-VI-d11 were purchased from Cayman Chemicals. Stock solutions were prepared in 100% ethanol and stored at -20°C. Chemicals used included: Butylated hydroxytoluene (BHT), Trolox and Glucose Oxidase, all obtained from Sigma Aldrich; Paraquat obtained from Acros organics and rotenone from Fluka.

### Cell culture and pro-oxidant treatment

The human hepatocellular carcinoma (HepG2) cell line and the human colon carcinoma cell line DLD-1 were both obtained from ATCC and DL-23 cells were generated from the DLD-1 line as described previously [13]. All cell lines were cultured at 37°C under a humidified 5% CO_2_ atmosphere in DMEM + GlutaMax containing 4.5 g/L glucose (Gibco), supplemented with 10% fetal bovine serum and 1% penicillin-streptomycin (Gibco). Experiments were performed in 9 cm tissue culture plates containing 10 ml of culture media. Pro- and anti-oxidants were added to media and incubated for indicated times before harvesting. 

### Sample preparation

Cells were harvested in ice-cold PBS and collected in a 1,5 ml centrifuge tube at 4°C and spiked with 2 ng deuterated internal standards before homogenization. Samples were homogenized using the Bullet Blender® (NextAdvance) with zirconium(VI)oxide (ZrO_2_) beads (0.5 mm), according to manufacturer’s instructions at 4°C. Homogenates were then hydrolyzed in 3 M KOH, containing 100 µM BHT [14] (3:1 v:v H_2_O:MeOH) for 45 min. at 45°C. Next, samples were cooled to room temperature and acidified to pH < 3 with HCl. Samples were then transferred to glass tubes and isoprostanes were extracted by vortexing with 5 ml ethyl acetate for 1 min., subsequently equilibrated on ice for 5 min., centrifuged for 5 min. at 3500g, 4°C, saving the organic phase for further solid phase extraction (SPE). NH_2_ Sep-Pak cartridges (Waters) were preconditioned with 5 ml hexane and subjected to a vacuum to obtain a flow rate of approximately 3 ml/min. An equal volume of ethyl acetate was used to rinse before elution with 3 ml ethyl acetate: MeOH: acetic acid (10:85:5 v:v:v). Eluates were dried under nitrogen and re-suspended in 150 µl 0.15% NH_4_OH: acetonitrile (90:10 v:v).

### Ultra-high performance liquid chromatography tandem mass spectrometry

Liquid chromatography was performed on an ACQUITY UPLC® system with a binary solvent manager (Waters). Isoprostanes were separated using an analytical BEH UPLC C18 column (150 mm x 2.1 mm, 1.7 µm particle size, Waters) at 40°C, over a linear gradient from 10-30% solvent B (solvent A= 0.15% NH_4_OH, solvent B= 95% acetonitrile: 5% MeOH, 0.0125% NH_4_OH) in 15 min. Column eluate was directly coupled to a Xevo™ TQ Mass spectrometer (Waters) fitted with an electron spray ionization probe operating in the negative ion mode with argon as collision gas. Cone voltage was set at 35 V, capillary voltage at 2.4 kV and desolvation gas temperature at 600°C with a flow rate of 750 (l/hrs). Multiple reaction monitoring (MRM) was used to analyze the various isoprostanes. F2-class VI isoprostanes were measured with a transition *m*/*z* 353>115 and F2-class III with transition *m*/*z* 353>193. Deuterated F2-class VI and III isoprostanes were measured with transitions *m*/*z* 362>115 and 357>197 respectively. Isoprostane concentrations relative to their labeled internal standards were determined using TargetLynx^TM^ (Waters) software. For each analysis, a fresh six point concentration series was prepared. 

### *In vitro* peroxidation assay

Arachidonyl PAF-C16 (Cayman Chemicals) was stored as a 5 mM stock solution in 100% ethanol under nitrogen at -20°C to ensure minimal auto-oxidation. For analysis, arachidonyl PAF-C16 was diluted to 5 μM in PBS, containing 50 μM CuCl_2_ and incubated at 37°C for indicated times in the presence or absence of 100 µM BHT. HepG2 cell homogenates were incubated for 7 hours at 37°C in the presence of CuCl_2_ (50 μM), either with or without BHT or Trolox (10 mM) followed by extraction and MS analysis.

### Quantification and statistical analysis

All data were normalized to the amount of protein per sample, using Bradford (BioRad) analysis according to manufacturer’s instruction. Experiments were performed in triplicate unless stated otherwise. Student t-test was used to test for significant differences between groups at p < 0.05. 

## Results

### F2-isoprostanes are formed from peroxidation of arachidonic acid

**Figure 1:**
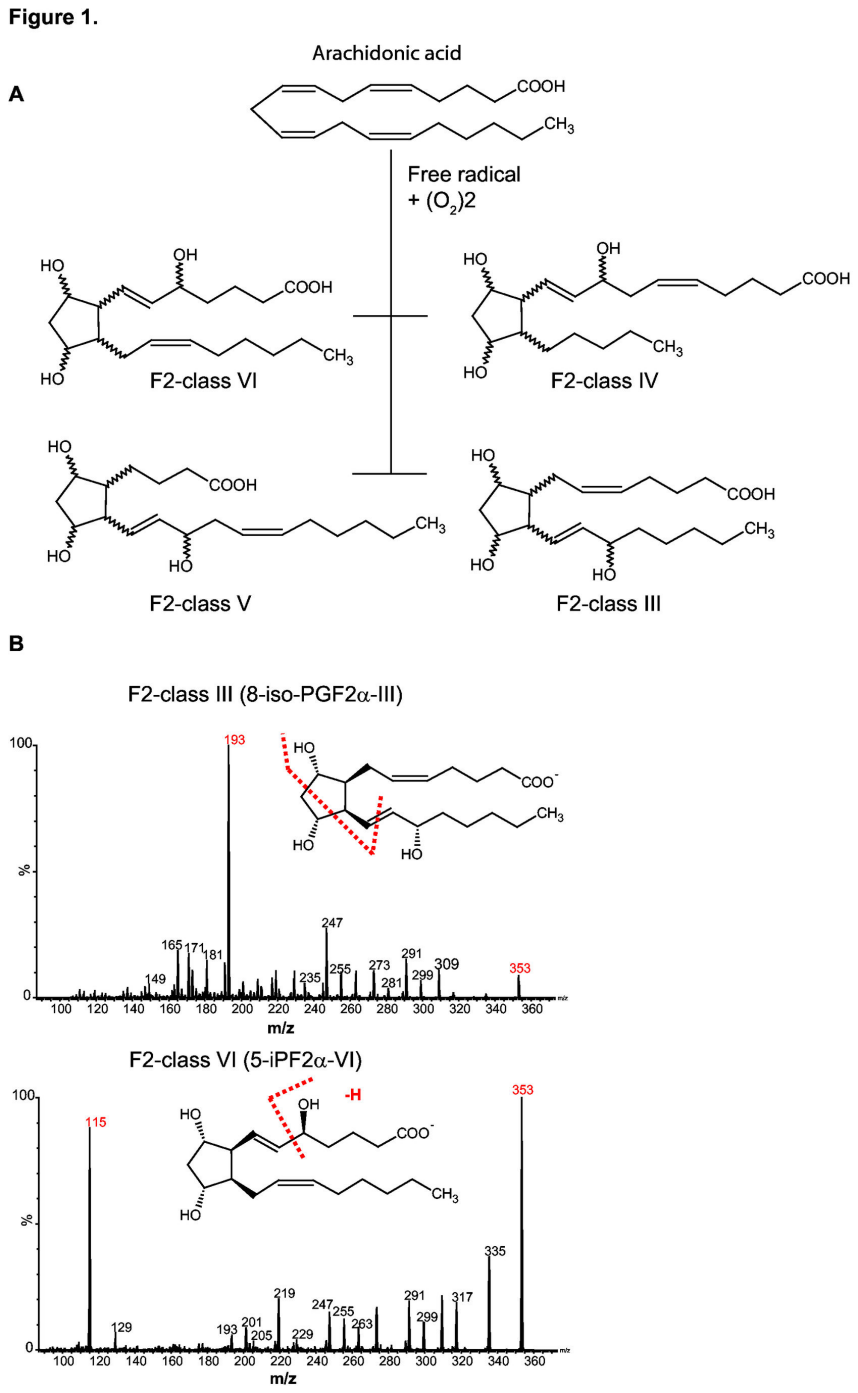
The chemical structure and mass spectrum of F2-isoprostanes. (A) Four isomeric classes of F2-isoprostanes are formed as a result of oxidative damage to arachidonic acid. (B) The product ion scan of F2-class-III and F2-class-VI isoprostanes. The ion masses indicated in red were used as MRM transition pairs for the indicated isoprostanes.

Arachidonic acid (AA) is a C20:4 omega-6 fatty acid and is the predominant polyunsaturated fatty acid (PUFA) in mammalian cells. It plays an important part in maintaining cell membrane integrity, fluidity and rigidity. It also acts as a precursor for eicosanoids, which are important signaling molecules. AA contains four double bonds, rendering it sensitive to ROS damage, which results in the formation of four F2-isoprostane isomer classes (Figure **1A**). Theoretically, each class can exist of 8 diasteriomeric forms for a total of 64 distinct isomers [15]. F2-isoprostanes are formed from AA esterified to phospholipids and to confirm this, we subjected arachidonyl PAF-C16, a glycerol-3-phosphocholine that contains AA to *in vitro* copper-induced lipid peroxidation and monitored F2-isoprostane formation using mass spectrometry. Copper-induced lipid peroxidation of arachidonyl PAF-C16 leads to the formation of lipid peroxyl radicals which initiate a chain of lipid peroxidation reactions resulting in F2-isoprostane formation [2,16,17].

**Figure 2:**
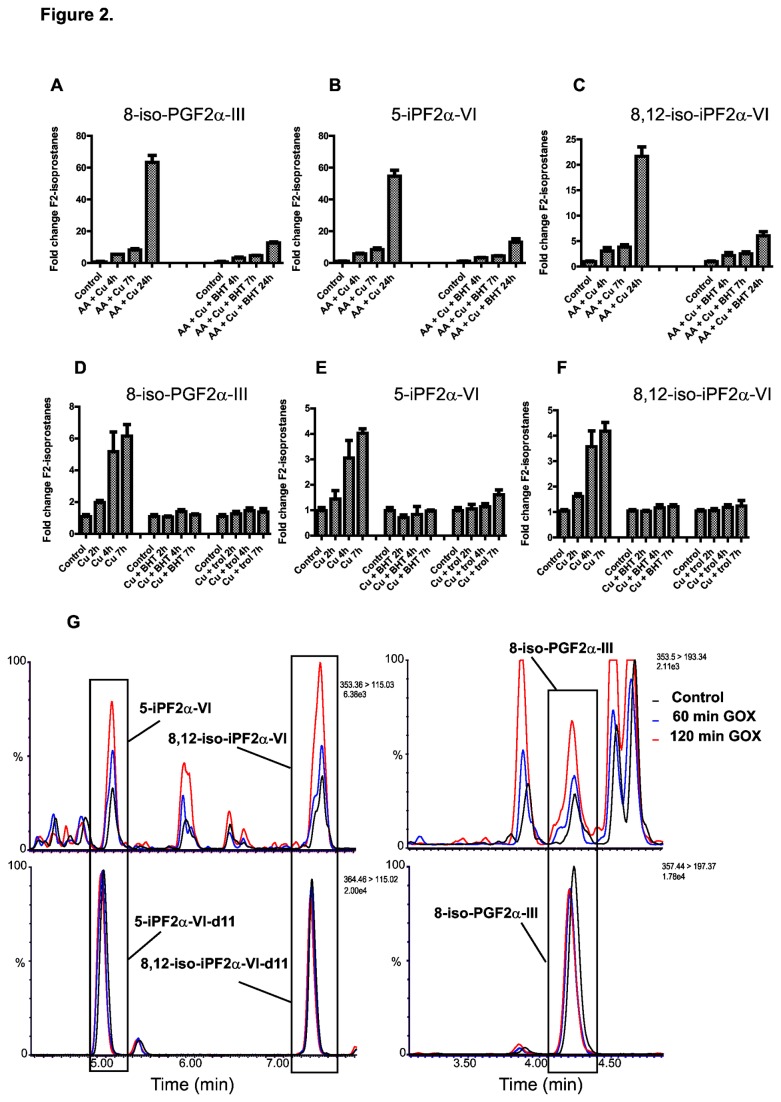
F2-isoprostanes are formed from peroxidation of arachidonic acid. (A-C) Copper-induced *in* *vitro* peroxidation of arachidonic acid resulted in the formation of three F2-isoprostane isomers. Arachidonyl PAF-C16 was subjected to 50 µM of CuCl_2_, with or without BHT, for the indicated time points and the levels of 8-isoPGF_2α_-III; 5-iPF_2α_-VI and 8,12-iso-iPF_2α_-VI were measured by UPLC-MS/MS. (D-F) F2-isoprostanes were formed in a time-dependent manner in cell lysates upon copper induced lipid peroxidation. HepG2 cell lysates were incubated with CuCl_2_ for indicated time points in the presence or absence of Trolox or BHT. Levels of 8-isoPGF2α-III; 5-iPF2α-VI and 8,12-iso-iPF2α-VI were analyzed in time by UPLC-MS/MS. Data are represented as mean ± S.D. (G) UPLC-MS/MS chromatogram overlay of extracted F2-isoprostanes of cells treated with GOX for indicated times.

Multiple Reaction Monitoring (MRM) was used to achieve optimum selectivity for the mass spectrometry analysis of the F2-isoprostanes. MRM monitors the precursor ion masses and, after fragmentation, the most predominant product ion masses. MRM transitions were determined by performing product ion scans for the F2-class-III and F2-class-VI isoprostanes (Figure **1B**). The MRM pair identified for endogenous 8-isoPGF2α-III was *m*/*z* 353>193 while it was *m*/*z* 353>115 for both 5-iPF_2α_-VI and 8,12-iso-iPF_2α_-VI. Although F2-class-VI isoprostanes have an identical MRM pair, they can be separated based on their unique retention times (Figure **S1**). Deuterated internal standards were used for absolute quantification. They have the same chemical properties and therefore the same retention times as endogenous F2-isoprostanes but with a higher *m/z* value, allowing for specific detection of F2-isoprostanes by co-elution. Their MRM pairs were determined in the same way as that of endogenous F2-isoprostanes and were found to be *m*/*z* 357>197 for 8-isoPGF2α-III-d4 and *m*/*z* 364>115 for both 5-iPF_2α_-VI-d11 and 8,12-iso-iPF_2α_-VI-d11. By using these MRM transitions in combination with their chromatographic retention and co-elution with their corresponding deuterated internal standards, we demonstrated the formation of three F2-isoprostane isomers including 8-isoPGF_2α_-III; 5-iPF_2α_-VI and 8,12-iso-iPF_2α_-VI upon *in vitro* peroxidation of AA (Figure **2A-C**). Note that an alternative isoprostane nomenclature is also in use [18]. These isomers were chosen since they are abundantly formed and synthetic standards are commercially available. There was a clear increase in the levels of all three isomers over time in response to copper treatment (Figure **2A-C**). This increase was blunted by the presence of butylated hydroxytoluene (BHT), an antioxidant that is known to terminate lipid peroxidation. It is interesting to note that all three isomers behave similarly in response to copper although, 8-isoPGF_2α_-III (Figure **2A**) showed the highest increase and 8,12-iso-iPF_2α_-VI levels (Figure **2C**) showed a lower response. To determine if these F2-isoprostanes are also formed in cells, lysates from the human liver carcinoma HepG2 cell line were prepared and subjected to copper-induced *in vitro* lipid peroxidation. Again, a clear response to the copper treatment for all the isomers was noted (Figure **2D-F**), whereas in the presence of BHT and Trolox, a water-soluble vitamin E derivative, there was no increase in F2-isoprostane levels (Figure **2D-F**). Next, isoprostane formation in living cells was tested by treating HepG2 cells with Glucose oxidase (GOX), which generates a constant flux of H_2_O_2_ in the presence of glucose and oxygen. Figure **2G** shows the overlay of the chromatograms of extracted F2-isoprostanes from cells treated for indicated times. It is clear that the areas under several peaks increased with increasing exposure time to GOX. These findings suggest that these peaks belong to endogenous F2-isoprostanes, which was confirmed by inclusion of the deuterated internal standards that were spiked to the sample after GOX treatment. Several peaks however that did respond to GOX, did not co-elute with the deuterated internal standards. Although it is possible that these belong to endogenous F2-isoprostane isomers, the absence of internal standards currently prevent definitive identification. Therefore, peroxidation of arachidonyl PAF-C16, cell lysates and living cells resulted in the formation of at least three F2-isoprostane isomers as markers of lipid peroxidation. 

### Method characterization, F2-isoprostane stability and metabolic fate in cells

**Figure 3:**
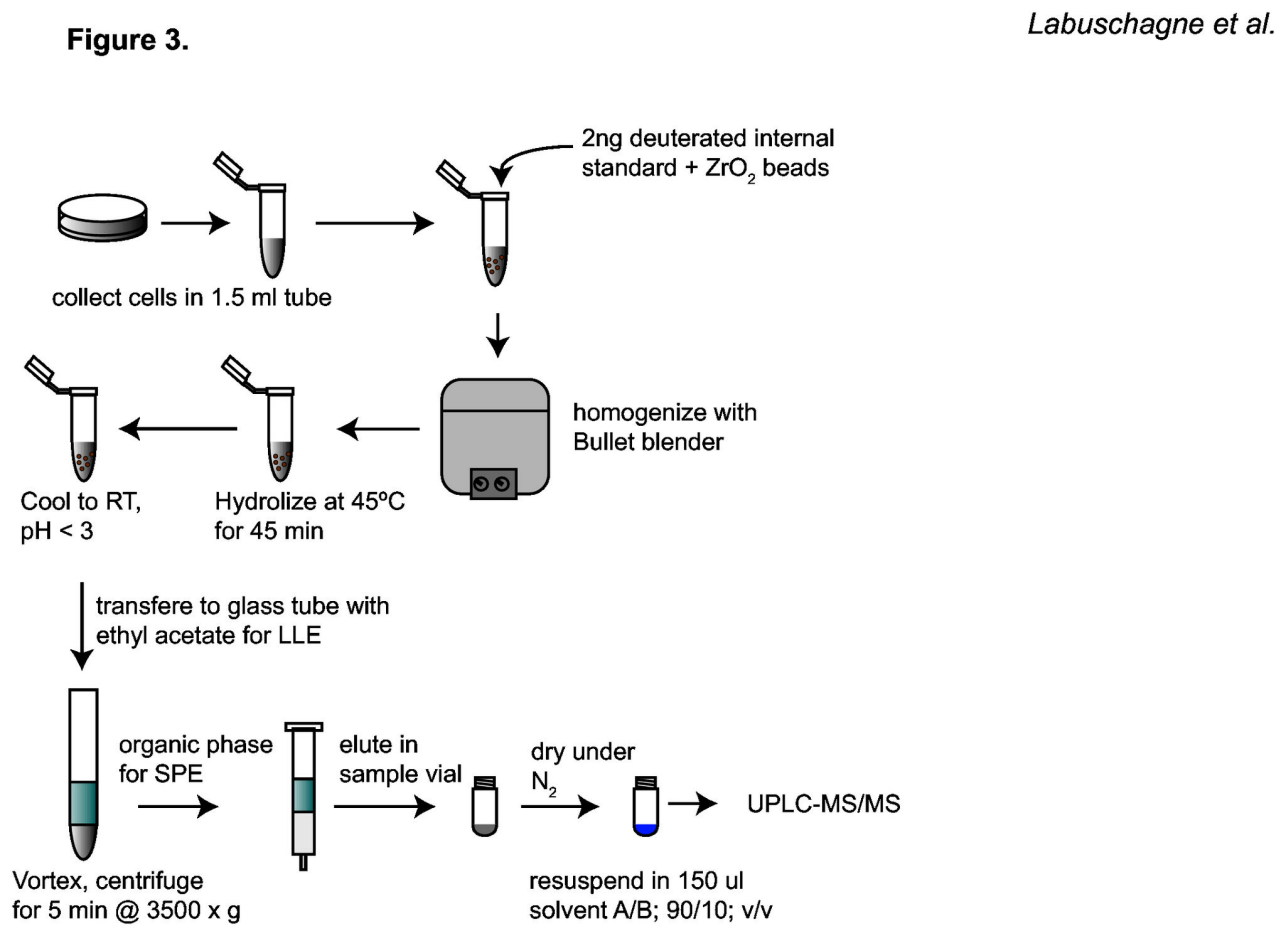
A schematic flow diagram of the sample preparation protocol for F2-isoprostane extraction from cultured cells. For detailed description, see materials and methods section. Abbreviations are: ZrO_2_ (zirconium(VI)oxide); RT (room temperature); LLE (liquid/liquid extraction); SPE (solid phase extraction).

**Figure 4:**
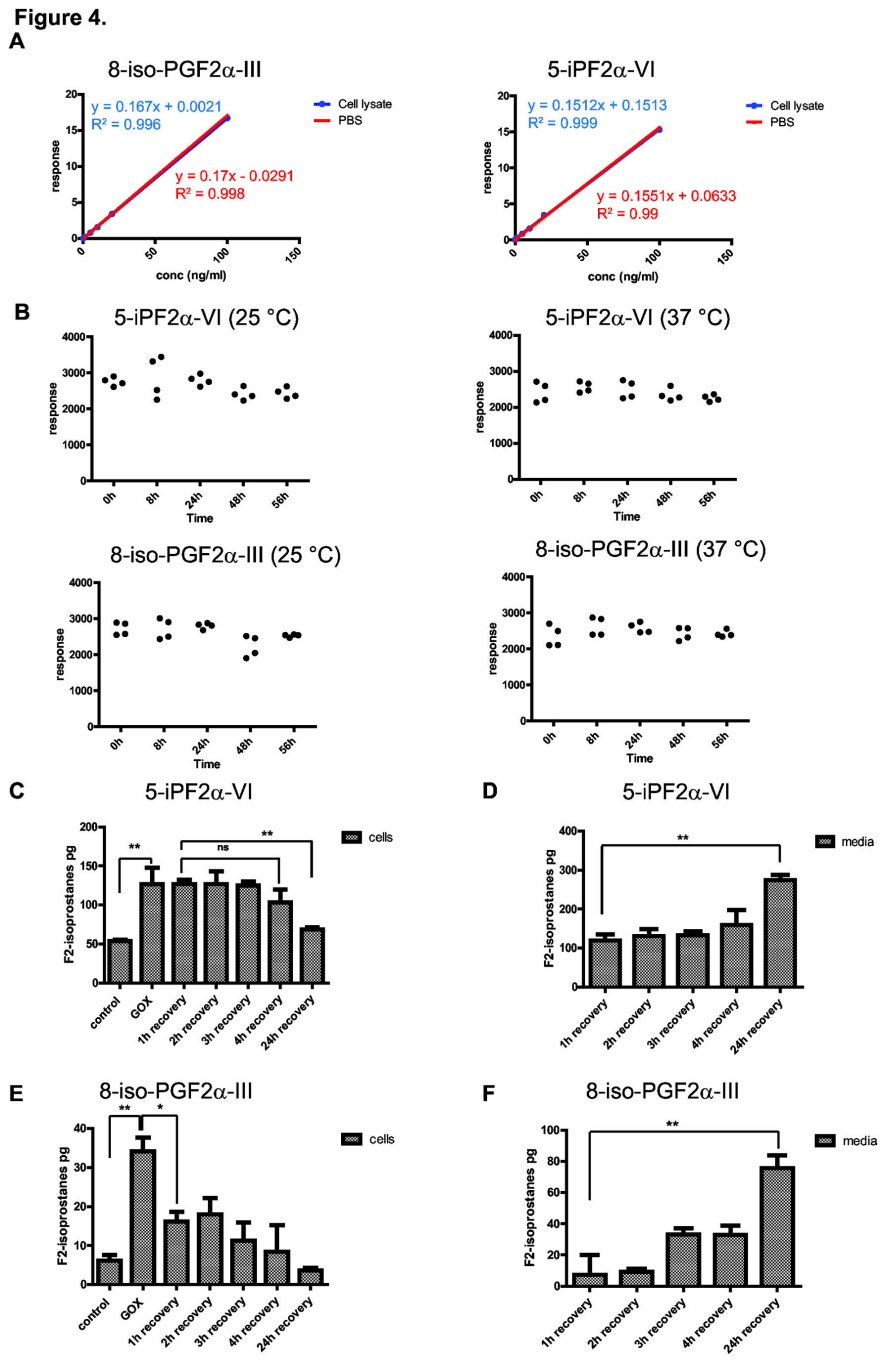
Characteristics of F2-isoprostane analysis in cells. (A) 8-isoPGF2α-III and 5-iPF2α-VI quantification is not affected by ion suppression. UPLC-MS/MS intensities of increasing concentrations of 8-isoPGF2α-III and 5-iPF2α-VI standards in PBS or cell lysates relative to a fixed amount (2ng) of their respective deuterated internal standard were plotted. (B) F2-isoprostanes are stable in physiological conditions. 8-isoPGF2α-III-d4 and 5-iPF2α-VI-d11 were spiked into a HepG2 cell lysate and incubated at 25 or 37°C for indicated time points. F2-isoprostanes were measured as in Figure 4A and scatter plots show individual data points. (C-F) 8-isoPGF2α-III and 5-iPF2α-VI are excreted in the culture media at different rates by HepG2 cells. HepG2 cells were treated with GOX for 2 hours to induce oxidative damage where after GOX was removed and cells were allowed to recover for indicated times. F2-isoprostane levels were monitored as in Figure 4A. Results are shown as mean fold increase ± S.D (* p < 0.05, ** p < 0.01,).

To characterize this protocol, synthetic and deuterated internal standards of two different F2-isoprostane isomers were used. Cell lysates are extremely complex as metabolite mixtures containing multiple molecules that potentially influence the extraction procedure of the isoprostanes as well as causing ion suppression in the source of the mass spectrometer, thereby affecting the analysis of F2-isoprostanes. We tested a range of extraction methods to optimize the protocol for cells and found that a combination of liquid/liquid extraction (LLE) and solid phase extraction (SPE) with aminopropyl (NH_2_) as stationary phase was optimal for F2-isoprostane detection in cells (Fig. **3**). A detailed description of this protocol can be found in the Materials and Methods section. To demonstrate that there is no matrix affect after extraction with this protocol, a series of cell lysates and PBS blanks were spiked with increasing concentrations of 8-isoPGF_2α_-III and 5-iPF_2α_-VI to compare the slopes of the fitted curves (Fig. **4A**). We show similar slopes of the curves for the two isomers and identified a linear response over a ten thousand fold dynamic range (Fig. **4A**). This finding confirmed that there is no effect of the metabolite matrix in cell lysates on extraction and ionization efficiency for the MS analysis of F2-isoprostanes using this protocol. Next, a cell number titration experiment was performed to establish the lower limit of the number of cells by which lipid peroxidation can be quantified. Using this protocol, 5-iPF_2α_-VI could be measured in as little as ten thousand cells with a signal to noise ratio (S/N) of 30 (Figures **S2A** and **S2B**). Furthermore, the limit of quantification (LOQ), calculated as a S/N of 10, was determined to be 1.5 pg/ml and the limit of detection set at S/N of 3 was 500 fg/ml. To the best of our knowledge, these are the lowest reported levels thus far. These findings suggest that the F2-isoprostane measurement is highly sensitive, allowing for the endogenous quantification of lipid peroxidation in as little as ten thousand HepG2 cells. Together, these results show that this UPLC-MS/MS stable isotope dilution assay, optimized for extracting and measuring F2-isoprostanes in cellular systems, allows for the detection and quantification of F2-isoprostanes in a robust and sensitive manner. The approach is furthermore linear over a wide dynamic range and, by making use of the deuterated internal standards, absolute quantification of F2-isoprostanes is possible.

One of the caveats of measuring oxidative damage is the fact that there is a constant turnover and repair of damaged molecules and these molecules might be chemically or metabolically unstable. In order to address this question for F2-isoprostanes, we spiked a HepG2 cell lysate with deuterated standards of 8-isoPGF_2α_-III and 5-iPF_2α_-VI, followed by longitudinal incubation at 25°C and 37°C to assess their stability under physiological conditions. We found that over a period of 56 hours there were no significant changes in the levels of the added deuterated standards for both isomers (Figure **4B**). This indicates that these two F2-isoprostane isomers are highly stable. To further determine the metabolic fate of the F2-isoprostanes we subjected HepG2 cells to 2 hours of GOX treatment to induce F2-isoprostane formation and monitored these levels in time. A threefold increase in F2-isoprostane levels was observed (Figure **4C**) and after removal of GOX from the medium, there was no significant decrease in F2-isoprostane levels in the cell lysate or an increase in excreted isoprostane levels in the culture media within the first 4 hours. (Figure **4C and 4D**). However, after 24 hours cellular F2-isoprostane levels were restored to that of baseline levels (Figure **4C**) and excretion was observed in the culture media (Figure **4D**). In contrast, the kinetics of the 8-isoPGF_2α_-III isomer were different. Already 1 hour after GOX removal, 8-isoPGF_2α_-III levels turned to baseline and excretion occurred more rapidly than for the 5-iPF_2α_-VI isomer, albeit at much lower levels (Figure **4E and 4F**). These findings suggest that 5-iPF_2α_-VI is more stable than the 8-isoPGF_2α_-III isomer in agreement with the notion that 8-isoPGF_2α_-III has been observed to be biologically active and subject to β-oxidation [15]. Therefore, we continued our analysis with the 5-iPF_2α_-VI isomer.

### F2-isoprostanes are formed in response to different ROS sources

**Figure 5:**
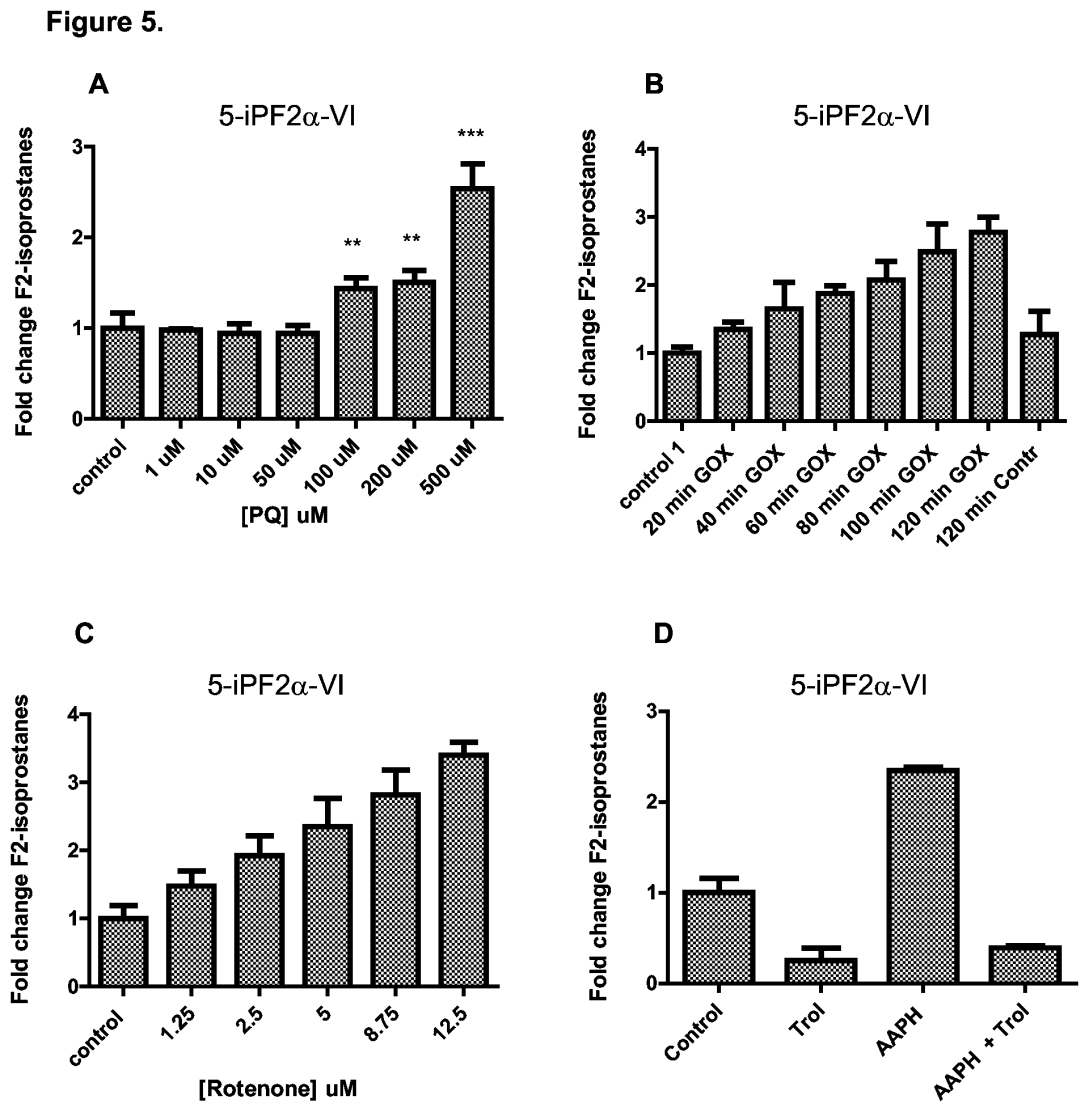
F2-isoprostanes are formed in response to different ROS sources. (A) F2-isoprostane levels increased in response to PQ treatment. HepG2 cells were treated with increasing concentrations of PQ for 24 hours. 5-iPF2α-VI levels were measured by UPLC-MS/MS. (B) Exogenous H_2_O_2_ exposure of HepG2 cells resulted in a 3-fold increase in levels of 5-iPF2α-VI. HepG2 cells were incubated in the presence of GOX for 2 hours and 5-iPF2α-VI levels were analyzed every 20 minutes. (C) Rotenone increased lipid peroxidation in HepG2 cells in a dose-dependent manner. HepG2 cells were treated with an increasing concentration of rotenone and F2-isoprostanes were measured after 24 hours. (D) Lipid peroxidation induced by the peroxyl radical generator, AAPH, is quenched by the antioxidant Trolox. Cells were treated with either Trolox, AAPH or both, for 24 hours. Data are shown as mean fold increase ± S.D over untreated control cells (** p < 0.01, *** p < 0.001).

To further explore the use of F2-isoprostanes as markers of oxidative damage in cellular systems, we subjected HepG2 cells to various pro- and anti-oxidants. Paraquat generates superoxide through redox cycling after take up by the cell, using NADPH as electron donor [19]. Cells were treated with different concentrations of PQ for 24 hours at 37°C and F2-isoprostane levels were measured. In cells exposed to concentrations lower than 100 µM PQ, no changes in 5-iPF_2α_-VI levels were observed. However, a significant increase in 5-iPF_2α_-VI levels was measured in cells treated with PQ concentrations equal to or above 100 µM and a more than 2 fold increase was observed after treatment with 500 µM compared to vehicle treated cells. (Figure **5A**). In contrast, when stimulated with 500 µM PQ in the presence of Trolox, there was no increase in 5-iPF_2α_-VI levels (Figure **S3**). These results show that PQ induced detectable cellular oxidative damage after treatment for 24 hours at concentrations ≥ 100 µM. Next, we subjected cells to an exogenous source of H_2_O_2_ by means of GOX treatment. Cells were harvested every 20 minutes for 2 hours and analyzed for levels of lipid peroxidation. There was a linear increase in 5-iPF_2α_-VI levels over time with a maximum response of about 3-fold induction (Figure **5B**). This suggests that lipid peroxidation increases over time in the presence of H_2_O_2_ in an accumulative manner. Since the mitochondria are considered as the main source of intracellular ROS, we treated cells with rotenone, a compound that generates ROS in the mitochondria by inhibiting electron flow in complex I in the electron transport chain (ETC). Rotenone treatment for 24 hours caused a dose dependent increase in 5-iPF_2α_-VI levels (Figure **5C**). This confirms that a blockage of electron flow in the ETC leads to an increase in ROS and ultimately to an increase in lipid peroxidation. The hydroxyl-radical (OH^•-^) is another critical ROS and is known to cause damage indiscriminately to DNA, proteins and lipids. The free radical generator 2,2’-azobis-2-methyl-propanimidamine dihydrochloride (AAPH) constantly produces OH^•-^ in solution during its decomposition [20]. A 2-fold increase in 5-iPF_2α_-VI levels was observed after 24 hours of AAPH treatment (Figure **5D**). Treatment with AAPH in the presence of Trolox did not result in an increase in lipid peroxidation. It is interesting to note that the control cells, treated with Trolox, in the absence of any ROS generator showed decreased 5-iPF_2α_-VI levels as compared to the untreated controls (Figure **5D**). This suggests that Trolox blocks endogenous ROS damage. Taken together, these results indicate that F2-isoprostanes are sensitive markers of cellular lipid peroxidation in response to multiple ROS.

### FOXO3a increase the resistance of cells against oxidative stress

**Figure 6:**
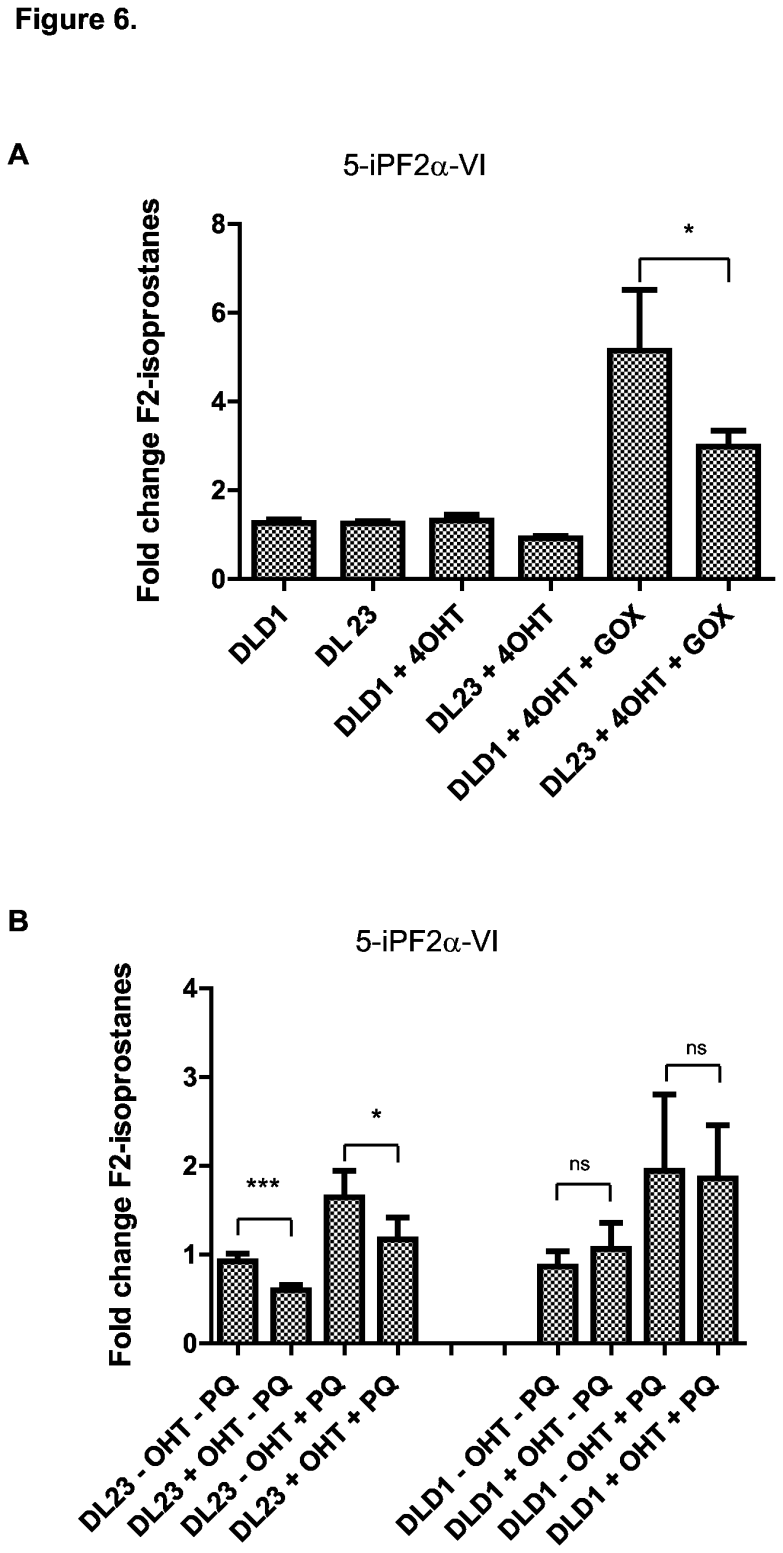
The F2-isoprostane approach allows for analysis of FOXO transcription factor control of endogenous oxidative stress. (A) GOX-induced lipid peroxidation is significantly decreased when FOXO3a is activated. DLD-1 and DL-23 cells were treated with 4-OHT to activate FOXO3a for 16 hours before H_2_O_2_ exposure by GOX treatment for 2 hours. F2-isoprostane levels were analyzed by UPLC-MS/MS and are shown as mean fold increase ± S.D (* p < 0.05) (B) Prolonged FOXO3a activation increased the protection against endogenously formed ROS damage in colon carcinoma cells. DLD-1 and DL-23 cells were incubated with 4-OHT to activate FOXO3a for 24 hours, before treatment with PQ for another 8 hours. 5-iPF2α-VI levels were analyzed and data are represented as mean fold increase ± S.E.M (*p < 0.05, *** p < 0.001).

Forkhead box O (FOXO) transcription factors are important regulators of cellular defense against ROS by controlling the gene transcription of ROS-scavenging enzymes, including SODs and catalases [21]. We employed two cell lines, DL-23 and DLD-1 to test the role of FOXO transcription of the cellular oxidative damage formation. The human colon carcinoma DL-23 cell line stably expresses a constitutively active FOXO3a construct fused to a modified form of the estrogen receptor hormone-binding domain which allows conditional activation of FOXO3a [22]. By adding the synthetic estrogen receptor agonist 4-hydroxy tamoxifen (4-OHT), the fusion protein is activated by entering the nucleus to initiate transcription [22]. The DLD-1 parental cell line, which stably expresses the empty vector, was used as control. The effect of FOXO3a activation on the stress resistance after treatment with GOX and PQ was determined using these cell lines. Firstly, both DLD-1 and DL-23 cell lines were treated with 4-OHT for 16h, after which cells were treated with GOX for another 2 hours to induce oxidative stress. There was a significantly higher increase in F2-isoprostanes in the DLD-1 cells as compared to the DL-23 cells after GOX treatment, which suggests that cells with activated FOXO3a are more resistant to exogenous oxidative stress. Although there was a trend for lower baseline levels of lipid peroxidation in the activated DL-23 cells, this did not reach significance (Figure **6A**). To determine if a longer activation time of FOXO3a could significantly affect baseline levels of lipid peroxidation and protect against endogenously formed ROS, cells were stimulated for 24 hours before treating them with PQ for another 8 hours. In this case, there was indeed a significant decrease in baseline F2-isoprostane levels in the DL-23 cells 24 hours after FOXO3a activation. These findings suggest increased ROS scavenging or repair capacity due to prolonged FOXO3a activation. Furthermore, the DL-23 cells had less PQ-induced oxidative damage as compared to the DLD-1 cells, indicating a FOXO3a-mediated protection against endogenous ROS damage (Figure **6B**). These results demonstrate that this UPLC-MS/MS protocol for measuring F2-isoprostanes in cells has the sensitivity to detect significant differences in the endogenous redox state controlled by transcription factor mediated scavenging capacity in cellular systems.

## Discussion

With the discovery of F2-isoprostanes as non-cyclooxygenase derived products of free radical- induced damage to AA-containing lipids [10], numerous studies showed them to be reliable markers of systemic oxidative damage in plasma and urine [15]. Here we present a protocol to accurately measure *in vivo* lipid peroxidation in cellular systems by using F2-isoprostane analysis. We show that at least three different F2-isoprostane isomers are formed in response to oxidative damage to an AA-containing phospholipid both *in vitro* and in living HepG2 cells. It is interesting to note that copper treatment induced the greatest response for the 8-isoPGF_2α_-III isomer as compared to the other two monitored isomers *in vitro* (Figure **2**). This observation is in agreement to the notion that there is a preference for the formation of different isomers during AA peroxidation [23]. However, when baseline levels of the F2-isoprostanes were measured in cultured cells 8-isoPGF_2α_-III levels were remarkably found to show the lowest concentration (Figure **4**). It is possible that 8-isoPGF_2α_-III is further metabolized in the cell, as rapid clearance and excretion was observed (Figure **4**). This is in accordance with studies which have shown that 8-isoPGF_2α_-III is subject to β-oxidation [15]. The isomer 5-iPF_2α_-VI however, showed a good response to *in vitro* peroxidation and baseline levels in cells were relatively high. No evidence thus far has been presented that shows that this isomer is further metabolized. This is in agreement with the high stability and efficient clearance that was observed (Figure **4**). Therefore, we propose to use this isoform as marker for oxidative damage in cellular systems. 

By using deuterated internal standard we were able to fully optimize and characterize this method in cells. By comparing the curve of an increasing concentration of F2-isoprostanes spiked in PBS and cell lysates, we could determine that the matrix had no effect on this extraction protocol of F2-isoprostanes and showed no ion suppression during mass spectrometry. We show that this method has a high dynamic range of ten thousand fold. It is extremely sensitive and we can measure 5-iPF_2α_-VI in as little as ten thousand cells with high confidence. Several technical papers have been published in previous years that describe isoprostane analysis by GC- and LC-MS. Although GC-MS technology is known to yield reproducible, robust and sensitive detection and quantification, the technology is limited in terms of throughput as it required time-consuming sample preparation and derivatization protocols [24]. LC-MS methods on the other hand are less time consuming but struggle to achieve GC-MS-like sensitivity [25,26]. Our protocol employs UPLC-MS/MS and through our optimized sample preparation procedure, high throughput studies to measure lipid peroxidation in cells during screening of compound libraries in the pharmaceutical industry are feasible. In fact, the lower limit of quantification reported here is the most sensitive reported of any GC- or LC-MS method to date. 

To validate this protocol, we subjected cells to different pro- and anti-oxidants and assessed oxidative damage through F2-isoprostane measurement. Levels of isoprostanes increased in response to different ROS species from different sources, including H_2_O_2_, OH^•-^ and •O_2_^-^. Although H_2_O_2_ is not a radical by itself, a Fenton reaction is induced in the presence of transition metal ions, which catalyze hydroxyl radical formation. As expected, there was a dose-dependent increase in oxidative damage in response to rotenone treatment. This demonstrated that this approach can assess damage originating from the mitochondria and more specifically from oxidative phosphorylation, which is considered the main source of baseline ROS and ROS damage in the cell. Interestingly, similar to rotenone, PQ also produces superoxide radicals, yet the kinetics of F2-isoprostane formation differed (Figure **5**). These findings might suggest that oxidative damage formation from superoxide produced by PQ treatment is distinct from the mitochondria. An attractive hypothesis would be that PQ mediates superoxide induction predominantly localized to cytoplasmic compartments. In line with this notion, PQ treatment was recently found to strongly induce oxidative damage in the nematode *C. elegans*, deficient in cytoplasmic SOD activity. In contrast, nematodes deficient in mitochondrial dismutase activity showed little response to PQ, which otherwise responded strongly to rotenone [2]. Further kinetic studies will be required to study the superoxide formation by PQ in detail.

The Forkhead box O transcription factors FOXO are important, conserved players in regulating cellular defense and repair mechanisms through transcriptional control of genes involved in ROS scavenging and DNA repair [27]. In addition to the study of the redox balance in cells in response to direct activators or inhibitors of ROS, an indirect system for redox analysis was used to test the sensitivity of the approach. By using an inducible FOXO3a expressing cell line, the endogenous redox response of transcription factor mediated redox control could be monitored, indicating that the F2iP protocol is sensitive enough to detect indirect redox control. 

Taken together, the 5-iPF_2α_-VI F2-isoprostane isomer is a stable marker for sensitive, accurate and linear quantification of endogenous lipid peroxidation in cellular systems, which could prove valuable for studies into the role of ROS and its damage in cells. This straightforward and short sample preparation protocol furthermore should allow high-throughput analysis. 

## Supporting Information

Figure S1 **A representative chromatogram of the MRM transition of F2-Class-VI isoprostanes demonstrating the chromatic separation of 5-iPF_2α_-VI and 8,12-iso-iPF_2α_-VI and co-elution of deuterated internal standards 5-iPF_2α_-VI-d11 and 8,12-iso-iPF_2α_-VI-d11.**
(EPS)

Figure S2 **Baseline levels of lipid peroxidation can be established in as little as ten thousand HepG2 cells.** (A) A titration curve showing the concentration of 5-iPF2α-VI for the indicated number of HepG2 cells. (B) A chromatographic representation of the signals of 5-iPF2α-VI measured in 1 x 10^4^ and 1 x 10^6^cells. The signal to noise ratios of 5-iPF2α-VI for 1 x 10^4^ and 1 x 10^6^ cells were determine to be 30 and 732 respectively. The red lines indicate the peak areas used for the determination of the signal to noise ratios.
(EPS)

Figure S3 **Trolox inhibit F2-isoprostane formation in cells treated with 500 µM PQ.** 5-iPF2α-VI levels were analyzed and data are represented as mean fold increase ± S.D.
(EPS)
